# Overexpressed circRANBP17 acts as an oncogene to facilitate nasopharyngeal carcinoma via the miR-635/RUNX2 axis

**DOI:** 10.7150/jca.55794

**Published:** 2021-05-19

**Authors:** Minghui Zhou, Puwen Zhang, Yulin Zhao, Rui Liu, Yujie Zhang

**Affiliations:** 1 Department of Rhinology, The First Affiliated Hospital of Zhengzhou University, Zhengzhou 450052, China.; 2 Department of Otorhinolaryngology, The First Affiliated Hospital of Zhengzhou University, Zhengzhou 450052, China.

**Keywords:** circRANBP17, miR-635, RUNX2, nasopharyngeal carcinoma

## Abstract

Circular RNAs (circRNAs) are implicated in the initiation and progress of several diseases, including cancer. However, the precise role of circRNAs in human nasopharyngeal carcinoma (NPC) remains unclear. In this research, we found a new circRNA hsa_circ_0001554 (circRANBP17), which was derived from the RAN binding protein 17 (RANBP17). Our qRT-PCR data found that circRANBP17 expression was up-regulated in NPC tissue and cells. Functional silencing studies revealed that circRANBP17 inhibited NPC cell proliferation and invasion *in vitro,* and circRANBP17 down-regulation also reduced tumor growth in nude mice. MiR-635 was demonstrated as a direct target of circRANBP17; circRANBP17 up-regulated RUNX2 expression levels by sponging miR-635, thereby promoting NPC proliferation and invasion. Thus, our data provide the evidence for the first time that circRANBP17 is a new onco-circRNA via miR-635/RUNX2 axis regulation, and may function as a novel therapeutic target for NPC treatment.

## Introduction

Nasopharyngeal carcinoma (NPC) is a highly aggressive head and neck squamous cell carcinoma, which may be caused by genetic, Epstein Barr virus, or environmental factors 1, 2. More than 70% of NPC patients are initially diagnosed with advanced locoregional disease due to disease aggressiveness, and a lack of specific symptoms 3. Similarly, complex pathogenesis and biological processes are key challenges facing the development of NPC therapeutics 4. Thus, better understanding of underlying pathological mechanisms is required to improve effective targeted therapies for NPC.

Circular RNAs (circRNAs) are endogenous non-coding RNAs (ncRNAs), generated by precursor mRNA back-splicing to generate continuously closed loop structures. Equally, they are also more resistant to degradation when compared with the linear RNAs 5, 6. Recent studies have supported that circRNAs are implicated in malignant tumor development and progression, including NPC 6. For example, Zhong *et al.* observed that the circular RNA, CDR1as promoted NPC cell proliferation and invasion by abrogating miR-7-5p induced E2F3 inhibition 7. Liu *et al.* also suggested that circSERPINA3 up-regulated MDM2 expression by acting on miR-944 to regulate NPC cell growth 8. Similarly, in their study, Hong *et al.* observed that circCRIM1 functioned as a competing endogenous (ceRNA) that promoted NPC metastasis, and docetaxel chemo-resistance via FOXQ1 up-regulation 9. However, the role of hsa_circ_0001554 (circRANBP17) in NPC remains unclear.

RUNX2 is a member of the Runt-related transcription factor (Runx) family, and is involved in various biologic processes, including tumor suppression 10. Runx inhibits c-Myc expression in a DNA-binding as well as C-terminal dependent way 11. Recently, RUNX2 was identified as playing critical roles in cancer progression; Li *et al.* revealed that elevated RUNX2 levels promoted breast cancer bone metastasis by enhancing integrin α5-mediated colonization 12. Colden *et al.* proposed that miR-466 impedes prostate cancer growth and bone metastasis, via RUNX2 regulation 13. Huang *et al*. observed that hsa_circ_0000144 boosted cell proliferation and invasion by regulating the miR-217/RUNX2 axis in bladder cancer 14. However, the precise function of RUNX2 in NPC cells remains unclear.

In present study, we observed that circRNA hsa_circ_0001554 (circRANBP17) significantly increased in NPC. Function assays showed that circRANBP17 promoted NPC proliferation and invasion *in vitro,* and increased tumor growth *in vivo*. Mechanistically, circRANBP17 appeared to act as a ceRNA of miR-635, directly targeting downstream RUNX2 and facilitating NPC progression. These data suggest that circRANBP17 may serve as a potential therapeutic target for NPC.

## Methods

### Patients and tissue

Thirty-eight (38) NPC tissues and 13 freshly frozen normal nasopharyngeal epithelial tissues were selected from the First Affiliated Hospital of Zhengzhou University. Washed in phosphate buffer saline (PBS) following collection, tissues were stored at -80 °C until required. Study protocols and research methods were reviewed and authorized by the Ethics Committee of our hospital, and were conducted according to the Declaration of Helsinki. All patients voluntarily provided the written informed consent.

### Cell culture and transfection

The human NPC cell lines, SNU46, SUNE1, CNE-1, HONE-1, CNE-2, HNE-1, and 6‐10B, and the human immortalized nasopharyngeal epithelial cell line (NP-69) were obtained from the Cell Bank of the Chinese Academy of Sciences (Shanghai, China). Cells were grown in RPMI-1640 medium (Gibco, New York, USA), 10% fetal bovine serum (FBS, Gibco) and 1% streptomycin/penicillin (Invitrogen, Carlsbad, USA). Cells were cultured in 5% CO_2_ at 37 °C.

A small interfering (si) RNA targeting circRANBP17 (si-circRANBP17#1: 3'-GAAATGAACCTGAGTTTGGCT-5'; si-circRANBP17#2: 3'-TCAGGAAATGAACCTGAGTTT-5'), miR-635 mimics, and inhibitors were purchased from Gene Pharma (Shanghai, China). Vectors and oligonucleotides were transfected into NPC cell lines by the use of Lipofectamine 3000 (Invitrogen, Waltham, MA, USA). Forty-eight hours later, transfection efficiencies were assessed by quantitative real-time PCR (qRT-PCR).

### RNA extraction and qRT-PCR

TRIzol reagent (Invitrogen, CA) was mixed with tissue or cells to extract total RNAs. We used PrimeScript RT reagent (Takara, Japan) for reverse transcription reaction, and SYBR Green Master Mix II (Takara) for PCR reactions. PCR was performed on an ABI 7900 fast real-time PCR system (ABI, CA). U6 or Glyceraldehyde-3-phosphate dehydrogenase (GAPDH) was employed as an endogenous control. Reactions were conducted three times, and data were analyzed using the 2^-ΔΔCT^ method. The following real-time PCR primers were used in this study: circRANBP17: Forward, 5'- CTCCAGGGTACTGTGGAACA-3' and Reverse, 5'-TCCAAGAGTGCTTTCTCAGC-3'; miR-635: Forward, 5'-AGTGCGTGTCGTGGTGT-3' and Reverse, 5'- GCCTGAGATGAAGCACGTG-3'; GAPDH: Forward, 5'-GGGAAACTGTGGCGTGAT-3', Reverse, 5'-GAGTGGGTGTCGCTGTTGA-3'; U6: Forward, 5'-CTCGCTTCGGCAGCACA-3', Reverse, 5'-AACGCTTCACGAATTTGCGT-3'.

### Subcellular fractionation

CircRANBP17 localization was evaluated using the PARIS™ Kit (Thermo Fisher Scientific). Briefly, cells were incubated in lysis solution, and then centrifuged for 3 min at 12,000 g. The supernatant and nuclear pellet were then used for cytoplasm RNA extracted, and qRT-PCR was conducted to assess circRANBP17 levels in the cytoplasm and nuclear fractions of NPC cells. 18S rRNA was chosen as a control for the cytoplasmic fraction, and U6 as a control for the nuclear fraction.

### Treatments with actinomycin D or RNase R

Actinomycin D is a common transcription inhibitor. Cells were incubated with 2 g/mL actinomycin D (Millipore, Billerica, MA, USA) for 0 h, 4 h, 8 h, 12 h and 24 h. Similarly, enzyme digestion analysis was performed with or without 3 U/mg ribonuclease R (Epicentre Technologies, Madison, WI, USA) in 2 μg total RNA for 30 min at 37 °C. After treatments, qRT-PCR was performed to detect circRANBP17 and RANBP17.

### Cell viability

At 24 h post-transfection, cells were placed in 96-well culture plates (3 × 10^3^ cells per well). Cell viability assays were then performed using the Cell Counting Kit-8 (CCK8; Dojindo, Japan) based on manufacturer's instructions. The absorbance at 450 nm was counted and recorded on a spectrophotometer.

### Colony formation

Processed cells were cultured in 6-well plates for 14 days to allow colony formation. After fixing in 4% paraformaldehyde, cells were stained in 0.1% crystal violet (Solarbio). Cell proliferation was estimated by the number of colonies with > 50 cells.

### Transwell assays

Cell invasion assays were conducted with cells (1 × 10^5^) cultured in Transwell upper chambers pre-coated with 2 mg/ml of matrigel (BD Biosciences, CA). In the top chamber, the medium was serum-free, whereas in the lower chamber, the medium was supplemented with FBS (10%). Approximately 24 h later, cells in the upper chamber were harvested with a cotton swab, fixed in methanol, stained in 0.1% crystal violet and counted under a microscope (Olympus).

### Luciferase reporter assay

Wild-type (Wt) and mutated (Mut) binding sites from miR-635 in the hsa_circ_0001554 sequence, or the RUNX2 3'-untranslated region (UTR) were sub-cloned into the pmirGLO reporter vector (Promega, Madison, WI), to construct circRANBP17-Wt/Mut and RUNX2-Wt/Mut. At 70% confluence, cells were transfected with either miR-635 or NC-mimics. After 24 h, luciferase activity was measured using a Luciferase Reporter Assay System (Promega).

### RNA immunoprecipitation (RIP) assay

RIP assays were performed using the Imprint® RNA Immunoprecipitation Kit (Sigma-Aldrich) as per manufacturer's instructions. Briefly, 1 × 10^6^ cells were lysed with RIP buffer and then incubated overnight with antibody-coated magnetic beads at 4 °C. Anti-immunoglobulin G (Anti-IgG) served as the reference for anti-argonaute-2 (Anti-Ago2). After RNA was isolated from the magnetic beads, qRT-PCR was conducted to analyze circRANBP17 and miR-635 expression levels.

### Tumor xenografts in mice

Ten 4 weeks old female BALB/c nude mice, purchased from Vital River Laboratory Animal Technology (Beijing, China), were divided randomly into two groups (3 mice per group). Approximately 2 × 10^6^ SUNE-1 cells, stably expressing down-regulated circRANBP1 (sh-circRANBP1), were subcutaneously injected into the right lower limbs of study mice. Tumor sizes were measured every week over a 5-week period. One indicator of tumor growth was tumor volume: i.e. (length × width2)/2. Seven weeks later, mice were sacrificed using a CO_2_ asphyxia method. Tumor tissue was then excised and weighed. This protocol was approved by the Animal Care and Use Committee of the First Affiliated Hospital of Zhengzhou University.

### Western blotting

Western blot assay was explored according to the previous study 12. Primary antibodies RUNX2 (anti-RUNX2, ab23981) were get from Abcam.

### Statistical analysis

All study data were analyzed using SPSS software (version 23.0) and presented as mean ± standard deviation (SD). We performed student's t-test between two groups, and one-way analysis of variance (ANOVA), followed by Tukey's test for more than three groups. A P-value < 0.05 was considered statistically significant.

## Results

### The up-regulation and identification of hsa_circ_0001554 in NPC

**Figure 1:**
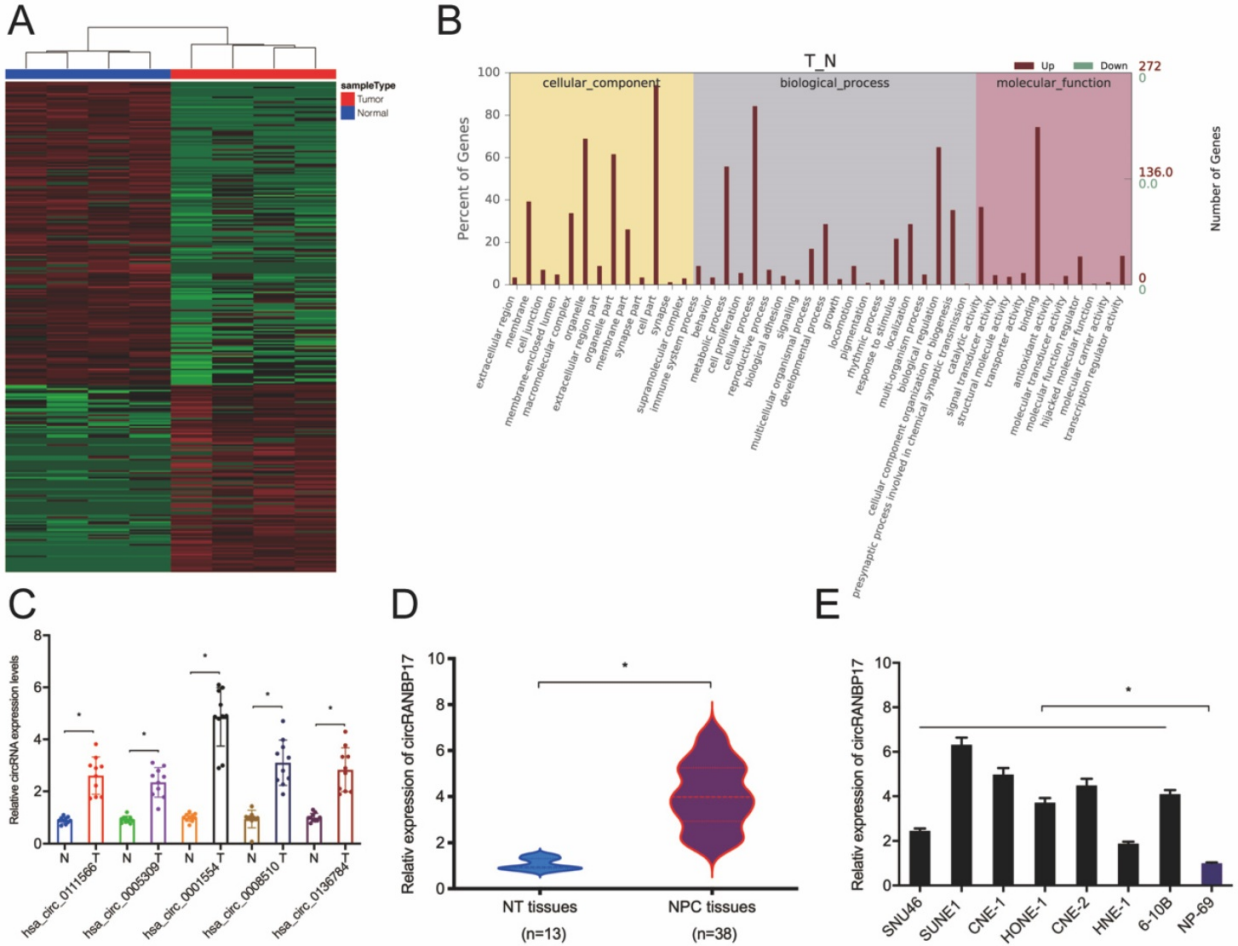
** circRNA profiles in NPC.** (A) A heatmap of circRNAs in four pairs of NPC tissue (GEO: GSE143797). (B) GO analysis of circRNAs in NPC and normal tissue. (C) qRT-PCR analysis was used to investigate the expression of the top five up-regulated circRNAs in NPC and normal tissue. (D) CircRANBP17 expression in NPC and adjacent normal tissue was investigated using qRT-PCR. (E) CircRANBP17 levels in NPC tissue and cell lines, i.e. SNU46; SUNE1; CNE-1; HONE-1; CNE-2; HNE-1; 6‐10B were measured by qRT-PCR. *P < 0.05.

Microarray datasets GSE143797 revealed a great deal of differential circRNAs between NPC and para-carcinoma specimens, according to the standard of values of |log2 FC| > 1 and P < 0.05 (Fig. 1A). Gene ontology (GO) analyses revealed differentially expressed circRNAs were involved in several physiological processes and cell signaling pathways, and had different molecular functions (Fig. 1B). Thus, we subsequently confirmed these microarray data using qRT-PCR. Five abundant circRNAs (hsa_circ_0111566; hsa_circ_0005309; hsa_circ_0001554; hsa_circ_0008510 and hsa_circ_0136784) were selected based on their significant differential expression. We finally selected hsa_circ_0001554 which exhibited significantly elevated differential expression (Fig. 1C). Next, we explored hsa_circ_0001554 (circRANBP17) expression in NPC tissues and cell lines; the qRT-PCR revealed that circRANBP17 level was significantly elevated in NPC tissues and cell lines (Fig. 1D and 1E).

**Figure 2:**
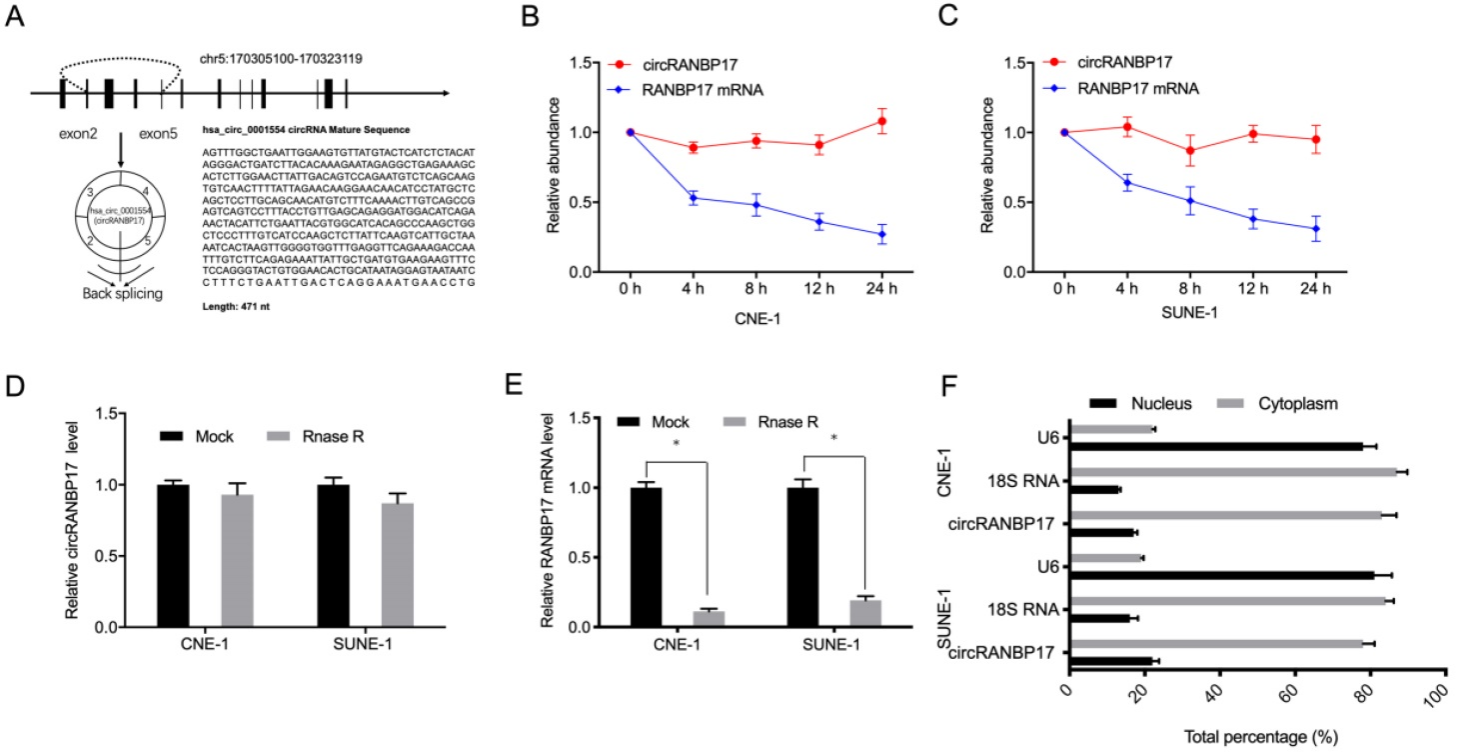
** circRANBP17 was highly expressed in NPC**. (A) The genetic location of hsa_circ_0001554 (circRANBP17). (B, C) circRANBP17 and RANBP17 mRNA levels were examined using qRT-PCR in NPC cells treated with actinomycin D. (D, E) The expression of circRANBP17 and RANBP17 mRNA in NPC cells treated with RNase R was measured using qRT-PCR. (F) CircRANBP17 expression in nuclear and cytoplasmic compartments in NPC cells. *P < 0.05.

Hsa_circ_0001554 (circRANBP17) is derived from exons 2-5 of the RANBP17 gene on chromosome 5:170305100-170323119. Its spliced mature sequence length is 471 base pairs (bp) (Fig. 2A). Next, to identify circRANBP17 as a circRNA, we used actinomycin D to treat NPC cells, and RNase R to digest isolated RNA from cells. Actinomycin D assays showed that circRANBP17 expression changed little, when compared to decreased RANBP17 in actinomycin D-treated NPC cells (Fig. 2B and 2C). RNase R assays revealed that this treatment degraded the RANBP17 linear transcript, but was ineffective towards circRANBP17 (Fig. 2D and 2E). Our subcellular localization analyses showed that circRANBP17 was predominantly localized to the NPC cytoplasm (Fig. 2F). Together, these data suggested that circRANBP17 overexpression was typical in NPC, which may elicit key functions in NPC development.

### CircRANBP17 knockdown reduces NPC progression

**Figure 3:**
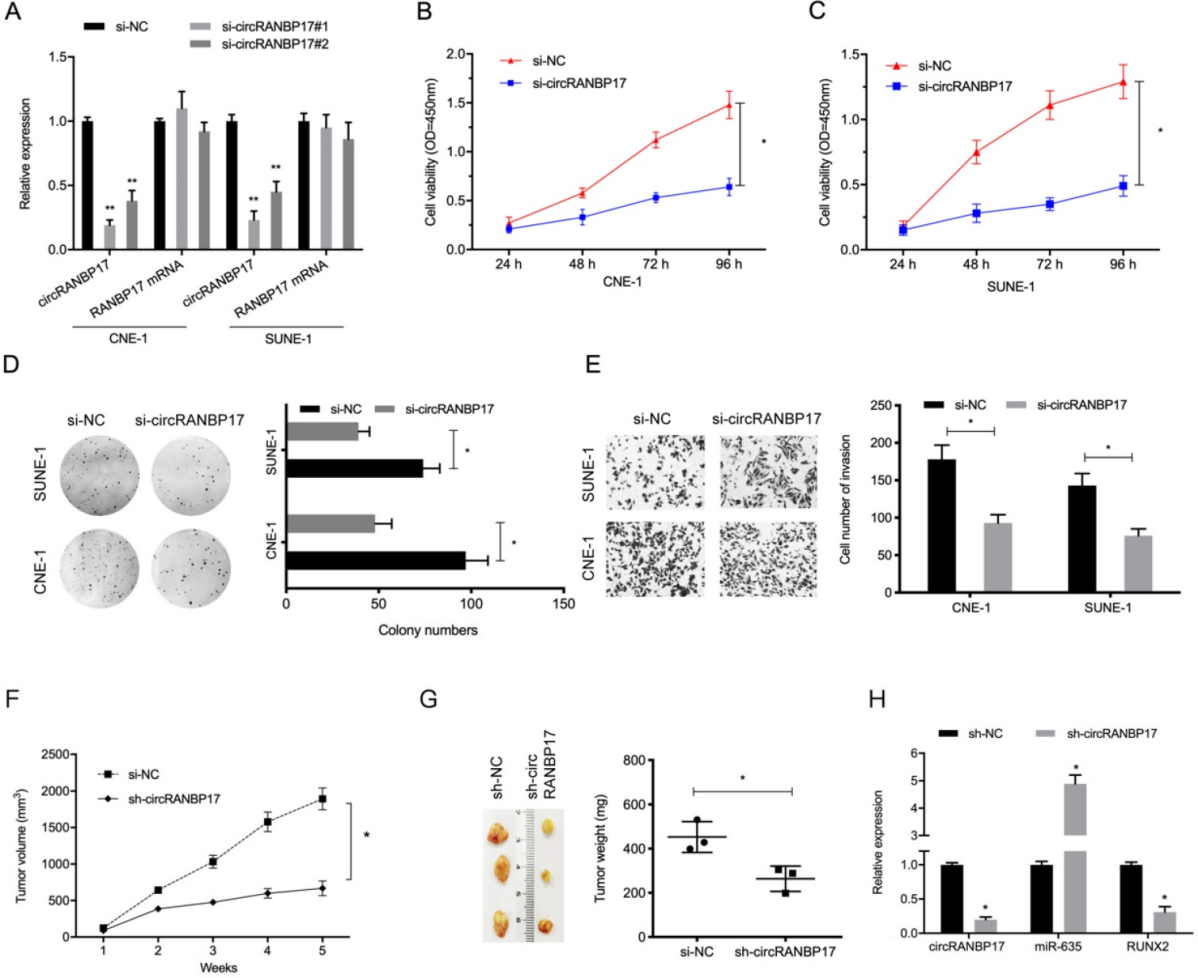
**circRANBP17 knockdown reduces NPC cell growth and invasion.** (A) The knockdown efficiency of circRANBP17 in NPC cells was verified by qRT-PCR. (B-D) CCK8 and colony formation assays revealed that circRANBP17 silencing reduced NPC cell proliferation. (E) NPC cell invasion was investigated using transwell invasion assay. (F-H) circRANBP17 inhibition reduced NPC cell growth *in vivo*. * P < 0.05.

To investigate the functions of circRANBP17 in NPC progression, si-circRANBP17 and si-NC were transfected into CNE-1 and SUNE-1 cells to assess circRANBP17 expression (Fig. 3A). The CCK-8 proliferation assay showed that circRANBP17 depletion inhibited cell viability in both cell lines *in vitro* (Fig. 3B and 3C). Similarly, colony formation assays demonstrated that colony numbers were decreased in both cell lines transfected with si-circRANBP17 (Fig. 3D). Additionally, cell invasion in the si-circRANBP17 groups was lower than the si-NC group (Fig. 3E).

Next, we tested whether sh-circRANBP17 exerted biological functions by promoting cell growth *in vivo;* RANBP17 suppression significantly reduced tumor growth in nude mice when compared to the control group (Fig. 3F-3H). These data indicated that circRANBP17 appears to promote growth in NPC.

### CircRANBP17 sponges miR-635 in NPC

**Figure 4:**
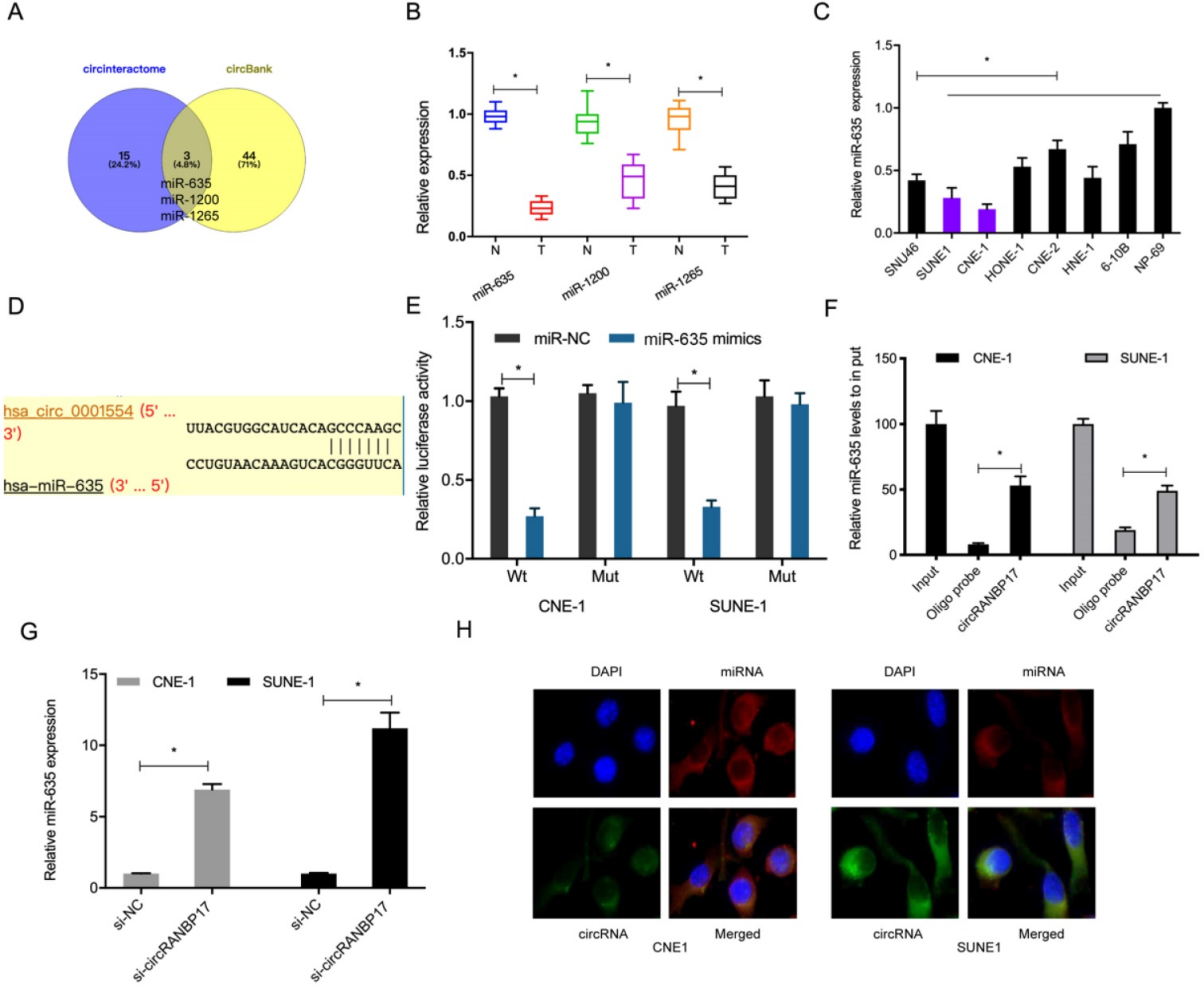
** circRANBP17 serves as a sponge for miR-635 in NPC cells.** (A) Potential target miRNAs of circRANBP17 were predicted using circBank and CircInteractome. (B, C) qRT-PCR was employed to assess the expression of miRNAs in NPC tissue and cell lines. (D) Schematic of circRANBP17 sequences at miR-635 binding sites. (E) The dual-luciferase reporter assay verified the binding of circRANBP17 and miR-635. (F) Pull-down assay verified miR-635 binding to circRANBP17 in NPC cells. (G) circRANBP17 suppression induced miR-635 expression in NPC cells. (H) FISH analysis of circRANBP17 and miR-635 in NPC cells. *P < 0.05.

Data from this study indicated that circRANBP17 was mainly localized to the cytoplasm, suggesting it could act as a miRNA sponge. To further investigate underlying mechanisms, we used the online databases, circBank and CircInteractome to explore potential miRNAs that may be sponged by circRANBP17. We identified miR-635, miR-1200 and miR-1265 (Fig. 4A). Of these miRNAs, miR-635 was significantly decreased in NPC tissues and cell lines (Fig. 4B and 4C), therefore it was chosen for further study. A dual-luciferase reporter assay presented that miR-635 mimics significantly decreased luciferase activity of circRANBP17-Wt, but not circRANBP17-Mut (Fig. 4D and 4E). A pulldown assay showed that endogenous miR-635 binding to circRANBP17 was specifically enriched by qRT-PCR analysis (Fig. 4F). Our qRT-PCR data showed that circRANBP17 silencing induced miR-635 expression levels in NPC cells (Fig. 4G). Moreover, fluorescence *in situ* hybridization (FISH) also showed that circRANBP17 and miR-635 were primarily expressed in the cytoplasm of NPC cells, suggesting sponging effects between them (Fig. 4H). Altogether, all these findings showed that circRANBP17 could act as a sponge for miR-635 in NPC cells.

### MiR-635 targets RUNX2 by binding to its 3'-UTR

**Figure 5:**
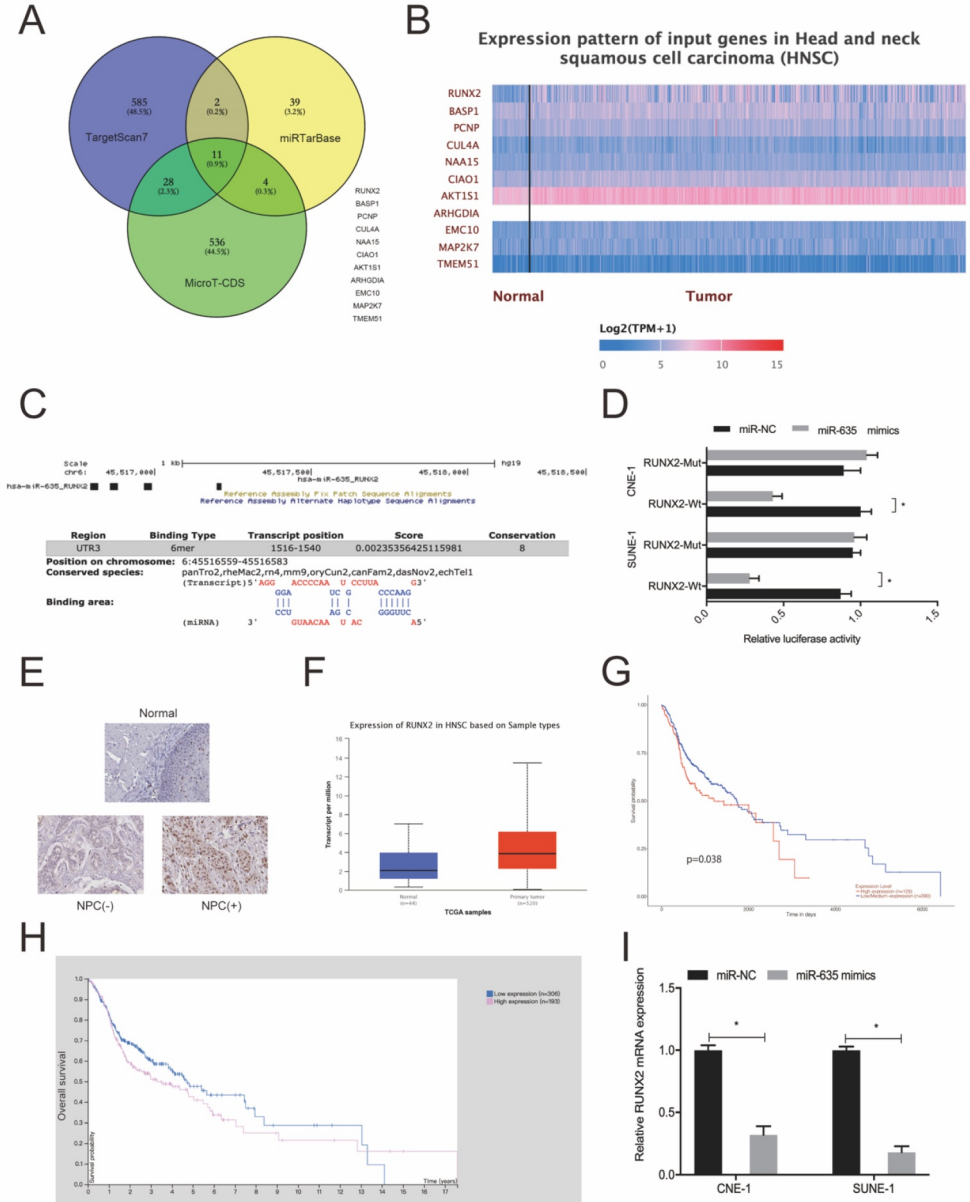
** miR-635 directly targets RUNX2.** (A) Potential miR-635 target genes were predicted by Targetscan, mirTarBase and MicroT-CDS. (B) Potential target gene expression as indicated by the TCGA database. (C) miR-635 binding sequences in the RUNX2 3`-UTR. (D) The dual-luciferase reporter assay confirmed the association between miR-635 and RUNX2. (E) RUNX2 expression in NPC tissue using IHC. (F) RUNX2 expression from the TCGA database. (G, H) Elevated RUNX2 expression was associated with poor overall survival in NPC patients. (I) MiR-635 overexpression decreased RUNX2 expression in NPC cells. *P < 0.05.

We used Targetscan7, mirTarBase and MicroT-CDS to investigate miR-635 target genes. Using intersection analyses, we identified 11 potential genes (Fig. 5A). Next, we explored the expression of these 11 genes using the TCGA database (Fig. 5B) and chose RUNX2 for subsequent study. We identified the potential binding site in miR-635 and the RUNX2 3'-UTR (Fig. 5C). A luciferase activity assay revealed that miR-635 mimics significantly impeded luciferase activity of the RUNX2-Wt 3'-UTR luciferase vector, whereas no effects were observed for the RUNX2-Mut 3'-UTR (Fig. 5D). Next, we explored RUNX2 expression in NPC tissue; our IHC data showed that RUNX2 expression levels were significantly increased in tumor tissues (Fig. 5E), which were further confirmed using the TCGA database (Fig. 5F). We also performed Kaplan-Meier analyses which showed that elevated RUNX2 expression levels were certainly associated with poor overall survival of NPC patients (Fig. 5G and 5H). Similarly, qRT-PCR showed that miR-635 overexpression decreased RUNX2 expression in NPC cells (Fig. 5I). These combined data showed that miR-635 targeted RUNX2 in NPC cells.

### CircRANBP17 promotes NPC growth by regulating the miR-635/RUNX2 axis

**Figure 6:**
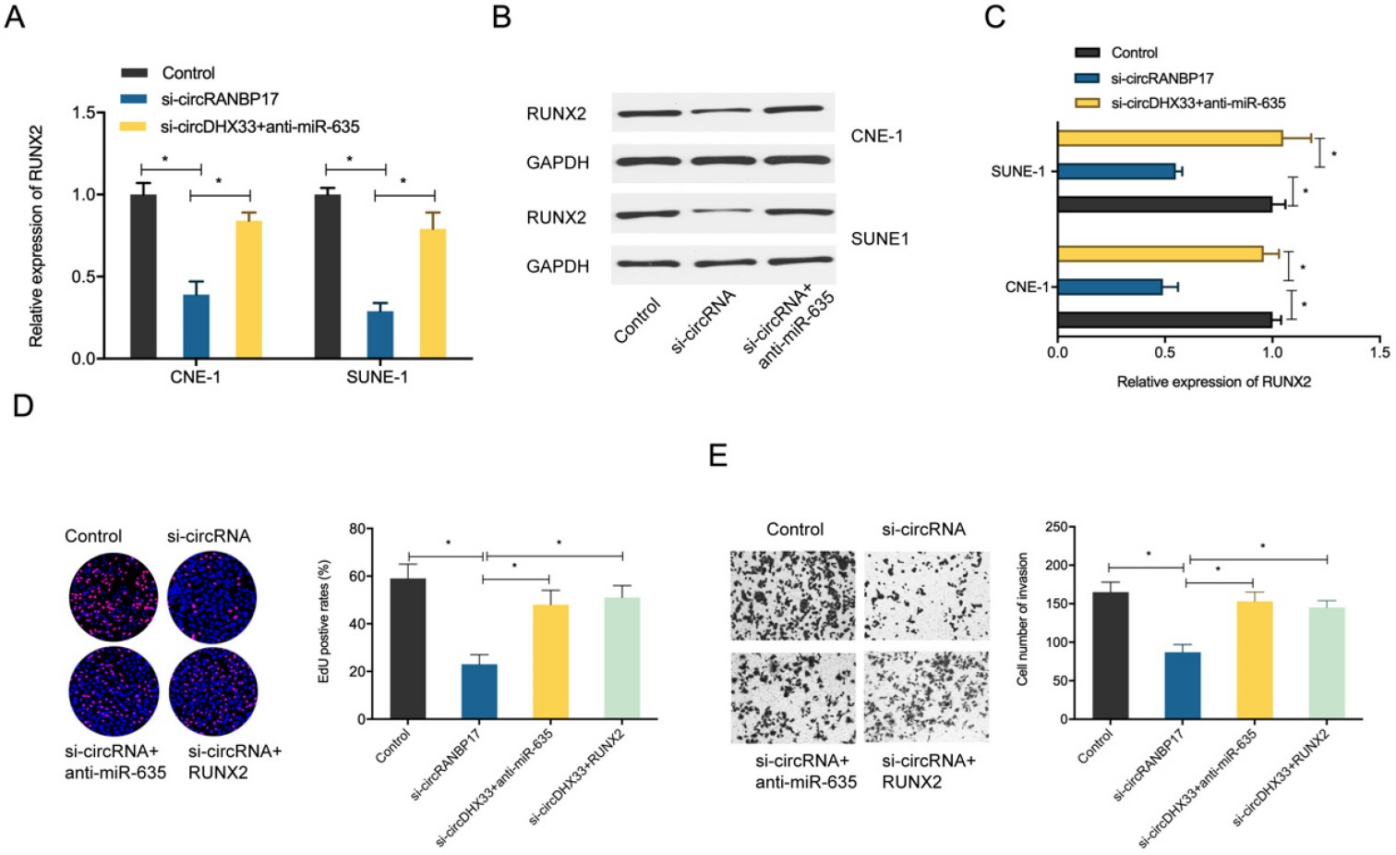
** circRANBP17 boosts NPC cell proliferation and invasion by elevating RUNX2 via miR-635 reduction.** (A-C) MiR-635 suppression reversed the role of circRANBP17 suppression on RUNX2 expression in NPC cells. (D) The EdU assay showed that miR-635 inhibitors or RUNX2 overexpression abolished the role of circRANBP17 suppression on NPC cell proliferation. (E) Transwell assay data showed that miR-635 inhibitors or RUNX2 overexpression abolished the role of circRANBP17 suppression on NPC cell invasion. *P < 0.05.

**Figure 7:**
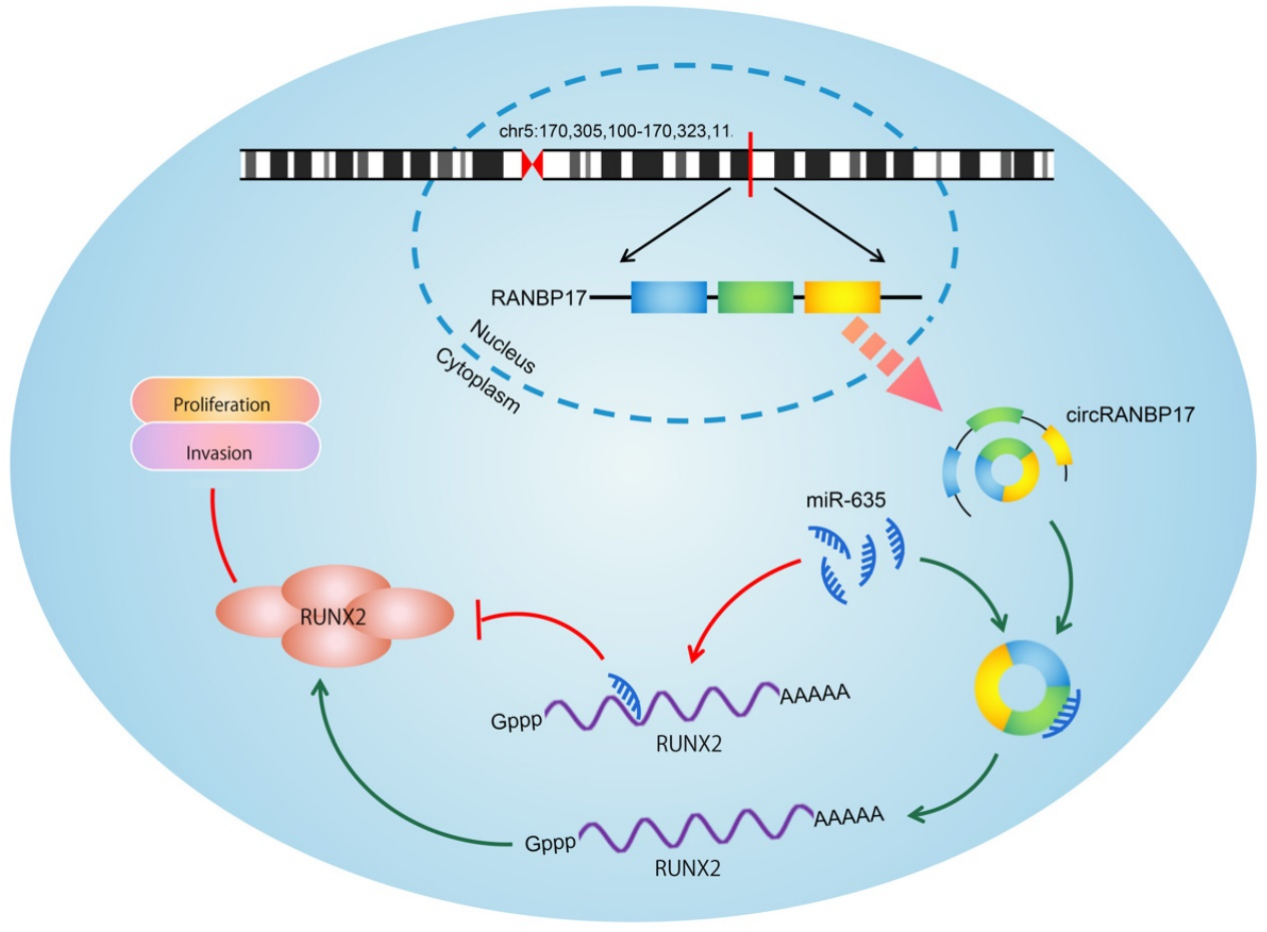
Schematic of the circRANBP17/miR-635/RUNX2 axis in NPC.

To determine whether the circRANBP17/miR-635/RUNX2 regulatory pathway was present in NPC, several assays were performed. We determined RUNX2 expression in NPC cells transfected with si-circRANBP17; our qRT-PCR and western blotting results indicated that si-circRANBP17 significantly reduced RUNX2 expression, while miR-635 inhibitors abolished the effects (Figure. 6A-6C). Next, we used rescue assays to further confirm the axis. EdU assay and transwell invasion assays revealed that miR-635 inhibitors (or RUNX2 overexpression) reversed the effects of circRANBP17 silencing in NPC cell proliferation and invasion (Fig. 6D-6E), suggesting that increased circRANBP17 reduced miR-635 activity to increase RUNX2 expression, thus promoting NPC cell proliferation and invasion (Fig. 7).

## Discussion

Studies have proved that circRNAs play crucial roles in diverse biological processes, including tumor genesis, metastasis and advancement 15, 16. Currently, several circRNAs have been associated with NPC progression, but the molecular mechanisms underpinning circRNA involvement in NPC progression remain unclear.

Using high-throughput sequencing analysis, we identified and characterized a novel circRNA, circRANBP17, which is back spliced from exons 2-5 of RANBP17. Interestingly, this gene was significantly overexpressed in our NPC tissues and cell lines. Next, we explored circRANBP17 in NPC progression, and revealed that circRANBP17 down-regulation blocked cell proliferation, invasion *in vitro*, colony formation and tumor growth *in vivo*, suggesting RANBP17 may have carcinogenic functions during NPC progression.

Increasing evidence has indicated that circRNAs principally serve as miRNA sponges to modulate a range of biological processes 17, 18. For instance, Shao *et al.* showed that hsa_circ_0001742 promoted tumor progression by acting as a sponge for miR-634 in tongue squamous cell carcer 19. Su *et al*. revealed that hsa_circ_0070269 reduced tumor progression in hepatocellular carcinoma via regulating the miR-182/NPTX1 axis 20. Wang *et al.* also found that circular RNA DHX33 promoted ccRCC cell growth by targeting the miR-489-3p/MEK1 axis 21. In this study, we discovered that circRANBP17 localization was primarily cytoplasmic in NPC cells, suggesting that circRANBP17 may exert influences via post-transcriptional regulation. Thus, we speculate that circRANBP17 may regulate NPC cell malignancy via miRNA sponge effects.

MiR-635 levels are decreased in some human tumors. Zhang *et al*. observed that miR-635 was less expressed in non-small cell lung cancer, and suppressed tumorigenesis 22. In their work, Cao *et al.* found that miR-635 targeted KIFC1 to inhibit gastric cancer progression 23. Zhu *et al.* observed that lncRNA PART1 promoted non-small cell lung cancer cell progression via regulating the miR-635/JAK-STAT axis 24. In this study, we showed that circRANBP17 may serve as a sponge for miR-635. Furthermore, the repressive effects of circRANBP1 knockdown on NPC cell malignancy were reversed by a miR-635 inhibitor. Hence, circRANBP1 appears to modulate NPC development via miR-635 sponging.

RUNX2 is a member of the mammalian Runt related transcription factor family, which have important roles in cancer development 25, however, precise RUNX2 functions and associated mechanisms in NPC remain unclear. In this study, we observed that RUNX2 was increased in NPC, and was a target of miR-635. In rescue assays, forced RUNX2 expression reversed si-circRANBP1 mediated inhibitory influences on NPC cell proliferation and invasion. Hence, these data suggested that circRANBP1 regulates NPC development by regulating the miR-635/RUNX2 axis.

In summary, our study is the first to demonstrate that circRANBP1 is an oncogenic circRNA in NPC, and facilitates cell proliferation and invasion by functioning as a ceRNA of miR-635 to increase RUNX2 expression levels. Hence, these data potentially provide a promising biomarker and therapeutic target for NPC.

## Abbreviations

NPC: Nasopharyngeal carcinoma
circRNA: Circular RNAs
miRNAs: MicroRNAs
Mut: Mutant
WT: Wild type
UTR: Untranslated region
